# Evaluation of a Trio Toscana Virus Real-Time RT-PCR Assay Targeting Three Genomic Regions within Nucleoprotein Gene

**DOI:** 10.3390/pathogens10030254

**Published:** 2021-02-24

**Authors:** Laurence Thirion, Laura Pezzi, Irene Pedrosa-Corral, Sara Sanbonmatsu-Gamez, Xavier De Lamballerie, Alessandra Falchi, Mercedes Perez-Ruiz, Remi N. Charrel

**Affiliations:** 1 Unité des Virus Émergents (UVE: Aix-Marseille Univ-IRD 190-Inserm 1207-IHU Méditerranée Infection), 13005 Marseille, France; laurence.thirion@ird.fr (L.T.); laura.pezzi3@studio.unibo.it (L.P.); xavier.de-lamballerie@univ-amu.fr (X.D.L.); 2 UR7310, Laboratoire de Virologie, Université de Corse-Inserm, 20250 Corte, France; falchi_a@univ-corse.fr; 3 Servicio de Microbiología, Hospital Universitario Virgen de las Nieves, Instituto de Investigación Biosanitaria ibs.Granada, 18014 Granada, Spain; irenee.pedrosa.sspa@juntadeandalucia.es (I.P.-C.); saral.sanbonmatsu.sspa@juntadeandalucia.es (S.S.-G.); Mercedes.perez.ruiz.sspa@juntadeandalucia.es (M.P.-R.)

**Keywords:** *Phlebovirus*, *Phenuiviridae*, meningitis, sand fly, *Phlebotomus*, arbovirus, Mediterranean, diagnostics

## Abstract

Toscana virus (TOSV) can cause central nervous system infections in both residents of and travelers to Mediterranean countries. Data mining identified three real-time RT-qPCR assays for detecting TOSV RNA targeting non-overlapping regions in the nucleoprotein gene. Here, they were combined to create a multi-region assay named Trio TOSV RT-qPCR consisting of six primers and three probes. In this study, (i) we evaluated in silico the three RT-qPCR assays available in the literature for TOSV detection, (ii) we combined the three systems to create the Trio TOSV RT-qPCR, (iii) we assessed the specificity and sensitivity of the three monoplex assays versus the Trio TOSV RT-qPCR assay, and (iv) we compared the performance of the Trio TOSV RT-qPCR assay with one of the reference monoplex assays on clinical samples. In conclusion, the Trio TOSV RT-qPCR assay performs equally or better than the three monoplex assays; therefore, it provides a robust assay that can be used for both research and diagnostic purposes.

## 1. Introduction

Toscana virus (TOSV) (species *Sandfly fever Naples phlebovirus*, genus *Phlebovirus*, family *Phenuiviridae*) is a negative-stranded, tri-segmented enveloped RNA virus transmitted by sandflies in the Mediterranean Basin [1]. Three genetic lineages have been identified to date, known as A, B, and C [2]. Lineage A has been reported so far in Italy, France, Turkey, Tunisia, and Algeria from both human cases and sandfly vectors [2,3]. Lineage B strains have been described in Portugal, Spain, France, Morocco, Croatia, and Turkey [2,3]. TOSV strains belonging to lineage C have been reported in Croatia and in Greece; although, the existence of this new lineage has been recognized based on partial sequences only, since the virus has not been isolated so far, nor characterized through complete genome sequencing [4,5,6].

TOSV is the etiological agent of central nervous system infections in both residents of and travelers to Mediterranean countries [3,7]. TOSV is also among the three most prevalent causes of acute aseptic meningitis in TOSV endemic areas [7,8]. The disease can be severe, with almost 50% of neurological forms including encephalitis manifestations; furthermore, permanent sequelae and fatal cases are rare but have been reported [2,9,10,11]. 

Despite the evidence of TOSV circulation in an expanding number of countries and its implication in neurological infections, there is little interest for this virus in the scientific community, and scarce knowledge among physicians on its symptoms spectrum [2]. Due to the lack of specificity of the clinical manifestations, laboratory diagnosis is necessary to differentiate TOSV infections from those caused by other etiological agents, and real-time RT-PCR is now considered the test of reference for diagnostic purpose at the acute stage of the infection [2,3]. 

The aim of this study was to perform data mining in order to identify peer-reviewed articles describing monoplex real-time RT-qPCR assays for detecting TOSV RNA, and to combine them to create a multi-region assay. Multi-region assays consist of the combination of two or more monoplex assays in the same reaction. The advantages of targeting different regions within the same gene have been recently described for SARS-CoV-2 [12]. A similar approach, consisting of targeting several regions in different genes, has been implemented for several pathogens such as human immunodeficiency virus (HIV), hepatitis B and C viruses (HBV and HCV), chikungunya virus (CHIKV), and Zaire ebolavirus (EBOV) [13,14,15,16,17,18,19,20]. In this study, (i) we evaluated in silico the three RT-qPCR assays available in the literature for TOSV detection, (ii) we combined the three systems to create the Trio TOSV RT-qPCR assay, (iii) we assessed the specificity and sensitivity of the three monoplex assays versus the Trio TOSV RT-qPCR assay, and (iv) we compared the performance of the Trio TOSV RT-qPCR assay with one of the reference monoplex assays on clinical samples.

## 2. Materials and Methods

### 2.1. Inventory of Published TOSV RT-qPCR Assays and Design of the Trio TOSV RT-qPCR Test

**Figure 1:**
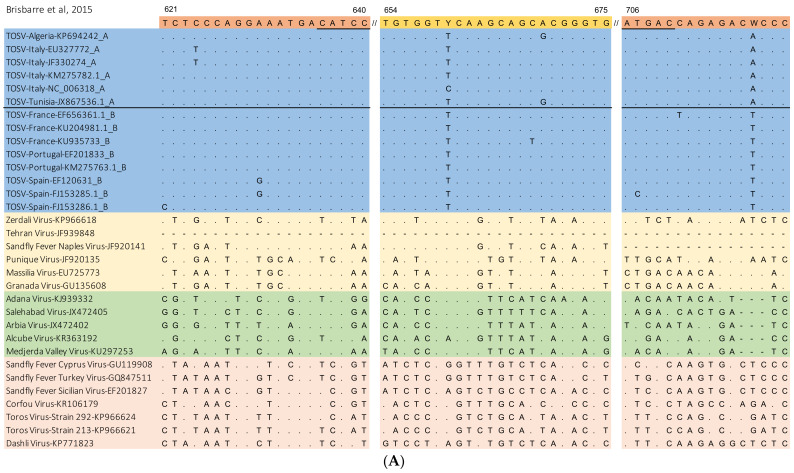

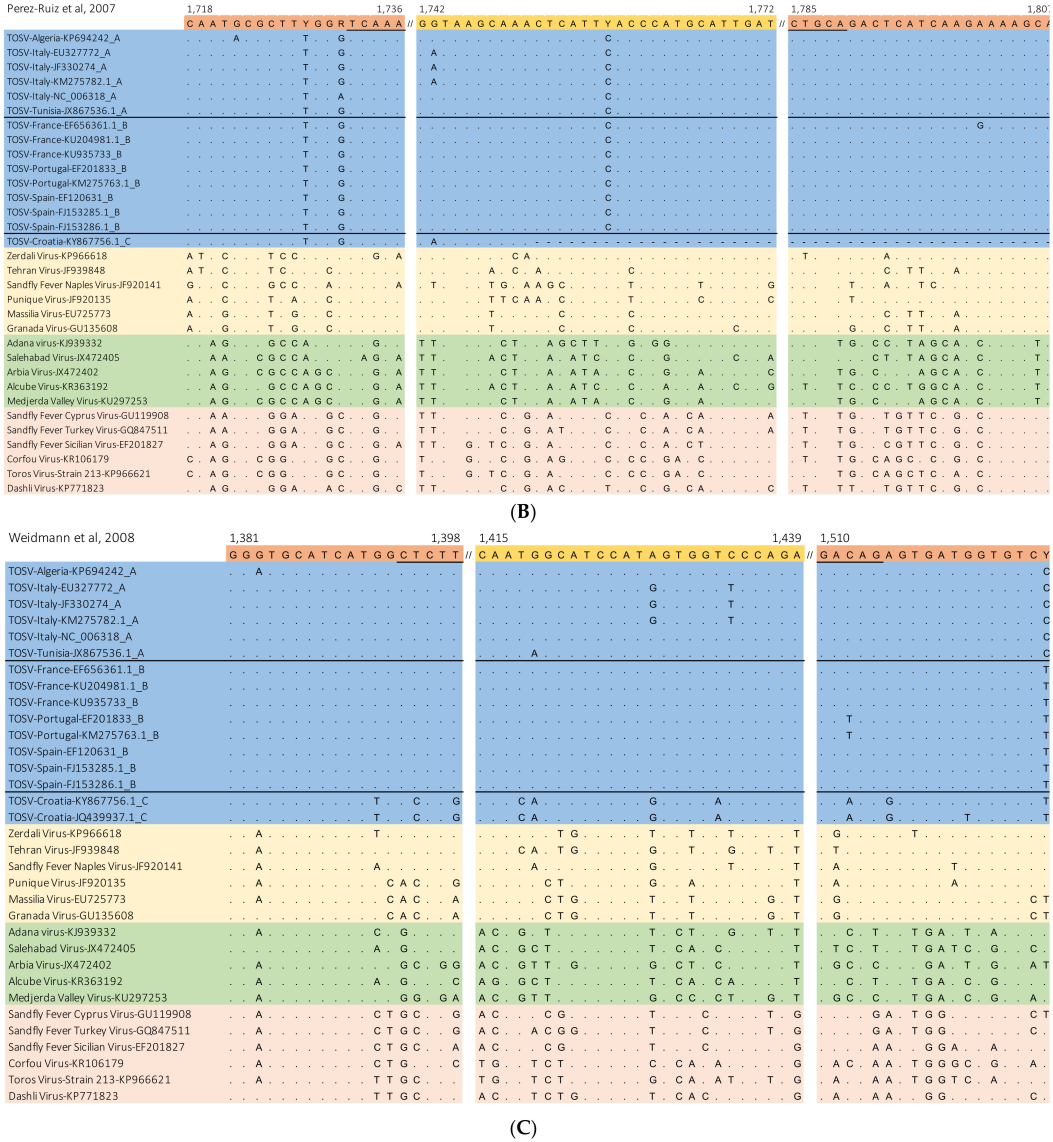
In silico analysis of the three RT-qPCR for TOSV detection included in the Trio TOSV RT-qPCR assay. In blue, TOSV strains; in yellow, viruses belonging to Sandfly Fever Naples virus serocomplex; in green, viruses belonging to Salehabad virus serocomplex; in orange, Sandfly Fever Sicilian virus serocomplex. TOSV genetic lineage (**A**–**C**) is indicated after the symbol “_” in the strain’s name.

TOSV RT-qPCR assays were searched for in NCBI PubMED using the search terms “Toscana”, “TOSV”, “polymerase chain reaction”, and “PCR” until April 2020. The three retrieved assays [21,22,23] were evaluated by in silico analysis using complete and partial sequences covering the S RNA region representative of the three TOSV lineages (6 sequences of lineage A, 8 of lineage B, and 2 of lineage C). Moreover, strains from phylogenetically close phleboviruses belonging to Sandfly Fever Naples virus, Salehabad virus, and Sandfly Fever Sicilian virus serocomplexes were added to the alignment. The list of sequences is available in Figure 1. The aim of this in silico analysis was to evaluate the adequacy of available TOSV assays for covering existing genetic variability among TOSV lineages, without detecting viruses other than TOSV. Sequence alignments were performed by using MEGA 7 software. Primers and probes of the systems were plotted against sequence alignment and evaluated for the number and position of mismatches as previously described [19,20,24]. 

### 2.2. RT-qPCR Assays

RT-qPCR reactions for the three monoplex assays and the “designed in this study” Trio TOSV RT-qPCR assay were performed with a SuperScript^®^ III Platinum^®^ One-Step RT-qPCR Kit with ROX (#11732-088, Invitrogen—Thermo Fisher Scientific, Waltham, MA, USA) on a BioRad CFX96TM thermal cycler, software version 3.1 (Bio-Rad Laboratories, Hercules, CA, USA). A volume of 5 µL of RNA was added to 20 µL of mix containing 12.5 µL of 2× Reaction Mix, 0.5 µL of Superscript III RT/Platinum Taq Mix, and primers and probes at the concentrations described in Table 1. Cycling conditions were: 50 °C for 15 min; 95 °C for 2 min; 45 cycles of 95 °C for 15 s; 60 °C for 45 s. All probes were labeled with the same dye (FAM). There were no modifications for neither the sequence nor the concentrations of the primers and probes of the three monoplex assays when combined in the same reaction tube. The only difference is that the quencher of the probe described by Pérez-Ruiz et al. has been modified to TAMRA instead of Dabcyl that was used in the original article. The reason for this is the need to have the same quencher for the probes of the three assays included in the Trio test.

### 2.3. Generation of RNA Synthetic Transcript (standard RNA)

An in-house synthetic standard RNA was used for the evaluation process of the Trio TOSV RT-qPCR assay, containing the three regions targeted by the three monoplex RT-qPCR assays included in the Trio assay. The standard RNA sequence is available in the Supplementary File. The three target regions, included in a plasmid synthesized by Genscript (GenScript, Piscataway, NJ, USA), were amplified by PCR. A first purification was performed using a Monarch PCR & DNA Cleanup Kit (New England BioLabs, Ipswich, MA, USA). The RNA transcript was synthetized in vitro by using a MEGAshortscript^TM^ T7 Transcription Kit (Invitrogen—Thermo Fisher Scientific, Waltham, MA, USA) according to the manufacturer’s instructions. TURBO DNase included in the same kit was used to remove any residual DNA. The RNA transcript was purified using a Monarch PCR & DNA Cleanup Kit (New England BioLabs, Ipswich, MA, USA). The RNA concentration was determined using a Thermo Scientific^TM^ NanoDrop^TM^ (Thermo Fisher Scientific, Waltham, MA, USA). The RNA transcript was serially diluted from 6 × 10^5^ to 10^2^ copies/mL, and dilutions were stored at −80 °C.

### 2.4. Sensitivity

The measure and comparison of the sensitivity of the three selected assays and of the Trio assay were performed using the RNA synthetic transcript, as well as two strains of TOSV, representative of lineages A and B. The TOSV strain belonging to lineage A (UVE/TOSV/2014/FR/5904) and the TOSV strain belonging to lineage B (UVE/TOSV/2013/FR/113) were both provided by European Virus Archive goes Global (EVAg, https://www.european-virus-archive.com/; references 001V-02452 and 001V-02461, respectively). Serial dilutions of the quantitated freeze-dried cell culture supernatant were prepared using AVE buffer containing 1 µg/mL of RNA carrier (QIAGEN, Venlo, The Netherlands) in order to achieve 5-fold serial dilutions containing 10^1^ to 6 × 10^5^ RNA copies/mL for both the RNA synthetic transcript and the TOSV strain from lineages A and B. Six decreasing concentrations were tested using twelve replicates for each. A Ct ≥ 40 was considered as negative. The lower limit of detection was determined by probit regression analysis using IBM SPSS software for standard RNA, TOSV A strain, and TOSV B strain. The LOD was defined as a concentration achieving a 95% positivity hit rate (LOD95). Detection rates of the Trio assay were compared to those of the three monoplex assays using a Chi-square test. 

### 2.5. Specificity 

The specificity of the Trio TOSV RT-qPCR assay was tested against several related and non-related viruses from *Phlebovirus* (n = 15)*, Alphavirus* (n = 7)*, Flavivirus* (n = 7)*, Enterovirus* (n = 3), and *Simplexvirus* (n = 3) genera. The panel includes 35 strains corresponding to phleboviruses distinct from TOSV, and to other viruses which co-circulate with TOSV or which can cause an overlapping clinical presentation with involvement of the nervous system. They are listed in the Supplementary File. All the viral strains included in the specificity panel were provided by European Virus Archive goes Global (EVAg, https://www.european-virus-archive.com/), except Lacrosse virus, which was provided by the National Collection of Pathogenic Viruses (NCPV, https://www.pheculturecollections.org.uk/collections/ncpv.aspx), and Varicella zoster virus, represented by a clinical sample (cerebrospinal fluid collected from a symptomatic patient).

### 2.6. Clinical Validation

Sixty-four cerebrospinal fluid (CSF) samples were included in this study. They were selected retrospectively based on the fact that, upon reception, they were found positive for the presence of TOSV RNA at the Microbiology Laboratory of the University Hospital “Virgen de las Nieves” in Granada, Spain. They had been stored at −80 °C during an eight-year period, between 10 July 2011 and 2 August 2019. 

The Trio TOSV reagents, in the form of Lyoph-P&P vials containing 24 reactions each (as previously described [25]), were sent to our Spanish partner who performed comparative analysis of Trio TOSV versus the assay routinely used in its laboratory for TOSV RNA detection [23]. The comparison was performed using two different Master mixes: (i) qScript™ XLT One-Step RT-qPCR (Quanta Bio, VWR International Eurolab S.L., Llinars del Vallès, Spain) and (ii) RealTime ready RNA Virus Master (Roche Diagnostics, Barcelona, Spain). The protocols are described in Table 2.

### 2.7. Ethics Statement

The study was developed in accordance with the Declaration of Helsinki. The clinical validation was a non-interventional study with no manipulation of clinical samples. Data of TOSV RNAs from clinical samples were retrieved from an anonymous database.

## 3. Results

### 3.1. TOSV RT-qPCR Assays and in Silico Analysis

Three TOSV RT-qPCR assays were retrieved from the PubMed search [21,22,23]. They were analyzed in silico, including strains of TOSV (lineages A, B, and C), as well as strains from phylogenetically close phleboviruses. Mismatches between this panel of sequences and primers and probe sets were checked, paying specific attention to the five 3’ terminal nucleotides of the primers, since mismatches in these positions can affect assay sensitivity. Results of the in silico analysis are presented in Figure 1.

Each of the three TOSV systems [21,22,23] consists of two primers and one dual-labeled probe and they amplify three non-overlapping fragments of the nucleoprotein gene (N) on the S segment. When compared with this panel of sequences, the three assays proved to have few mismatches, generally not located in critical positions, with TOSV strains belonging to lineages A and B. Just two partial sequences are available in GenBank for detection of the S segment of TOSV lineage C; both fully cover the target of the Weidmann assay, and one partially covers the target of the Pérez-Ruiz assay (1718–1751 nt). Comparison with the few available genetic data on lineage C showed one possible mismatch with the probe of the Pérez-Ruiz assay, while several mismatches were observed within the primers and probe of the Weidmann assay. Nevertheless, we decided to include both assays in the Trio TOSV RT-qPCR with no modifications for three reasons. Firstly, the degeneration of the Weidmann assay could have led to a loss of sensitivity in the detection of TOSV strains belonging to lineages A and B. Secondly, the Pérez-Ruiz assay is the one used to detect the three TOSV strains of lineage C identified so far, so its presence in the Trio TOSV RT-qPCR without modifications ensures detection of lineage C strains even in cases of failed detection by the Brisbarre and Weidmann assays. Thirdly, the two available sequences of lineage C could not be representative of the whole C lineage. The three assays provided a high number of mismatches with related phleboviruses, proving to be highly specific for the detection of TOSV.

### 3.2. Specificity of Trio TOSV RT-qPCR Assay

Trio TOSV RT-qPCR detected the two TOSV strains (one belonging to lineage A, the other one to lineage B) used as positive controls and did not react with the 35 viruses included in the specificity test panel, proving to be specific at 100% for TOSV. 

### 3.3. Sensitivity

**Figure 2:**
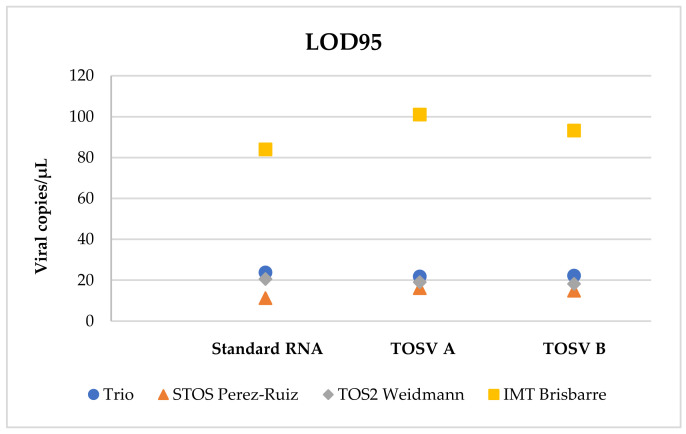
Comparison of the limit of detection 95% (LOD95).

LOD95 of the Trio TOSV RT-qPCR assay was 23.8 viral RNA copies/µL for standard RNA, 21.9 viral RNA copies/µL for TOSV A strain, and 22.3 viral RNA copies/µL for TOSV B strain (Table 3). Detection rates of the Trio TOSV, Pérez-Ruiz and Weidmann assays were not significantly different for standard RNA and TOSV A and B strains. Trio TOSV performed significantly (*p* < 0.05) better than the Brisbarre assay in the detection of both standard RNA and TOSV A, but not for TOSV B (Figure 2). 

### 3.4. Clinical Validation

All 64 TOSV RNAs from CSF samples were tested using the QuantaBio Master mix, whereas only 51 could be tested with the Roche Master mix. Of the 64 samples tested with the QuantaBio Master mix, 61 were found positive with both assays (Trio TOSV and Pérez-Ruiz monoplex assay [23]) with no discrepancies using qualitative criteria (positive/negative). When quantitative analysis was performed using the Ct value, the Trio TOSV assay showed lower Ct values in 51 out of 61 tests with an average reduced Ct value of 1.55. In contrast, Ct values of specimens #52 and #53 were much higher with the Trio TOSV assay (44.5 vs. 31.9 and 41.1 vs. 28.4). This difference can be explained by a technical problem, although there is no evidence for or against this hypothesis, and no more material was available for repetition. As concerns the three samples found negative with both the Trio TOSV and the monoplex assay using the QuantaBio Master mix, two were also negative with the Roche Master mix, and the sample volume was insufficient to test the third sample with the Roche Master mix. Accordingly, it is highly probable that degradation of the RNA due to freeze/thaw cycles can be the cause of the discrepancy between the original results and those observed in our study.

With the Roche master mix, 49 out of 51 samples were found positive by both assays (Trio TOSV and Pérez-Ruiz monoplex assay [23]): 38/49 samples provided a lower Ct value with Trio TOSV, although the average ΔCt value was not as high as observed with the QuantaBio Master mix (0.2 Ct). The detailed results of molecular testing on clinical material are presented in Supplementary File.

## 4. Discussion

We developed and evaluated a Trio RT-qPCR assay for the detection of TOSV RNA, by combining three monoplex assays with different targets on the viral genome. The advantages of targeting more than one region of the genome for diagnostic purposes have been extensively described in previous studies [16,26,27,28,29,30]; specifically, compilation of those advantages can be found in the most recent studies [19,20,24]. Therefore, they were not developed again here. 

As previously underlined, the advantage of targeting more than one region of the genome is to prevent false-negative results caused by mutations often observed with emerging pathogens, especially with RNA viruses since they have high evolutionary rates compared to DNA pathogens [31]. Moreover, using a multi-region assay is particularly useful for the detection of pathogens for which there is limited available sequence data, giving way to possible mispriming in yet to be identified genetic variants. This is exactly the situation encountered with TOSV for which there is a limited number of sequence data compared with other arboviruses such as dengue, yellow fever, West Nile, Zika, chikungunya, or tick-borne encephalitis viruses. 

Here, we decided to develop a multi-region assay that combines three monoplex assays in the same reaction tube rather than two as we did for our Duo CHIKV and SARS-CoV-2 RT-qPCR assays [19,24]. This choice is explained by the fact that several gaps of knowledge exist for TOSV: only three lineages are actually known, with very few genetic data available for the recently detected lineage C; moreover, we ignore if other lineages, undetected so far, exist. The presence of three monoplex assays in the Trio TOSV RT-qPCR increases the probability of detecting future TOSV strains and eventually new lineages, since the possible failure of one system can be compensated by the other two assays. We are well aware that detection of TOSV RNA will soon benefit from increasing sequence data that will enable the development of alternative assays located in other genome segments. It is, therefore, highly plausible that the Trio TOSV assay will help to detect new cases, subsequently increasing the mass of genomic data, mandatory to design an even better RT-qPCR assay. 

The Trio RT-qPCR assay includes three monoplex assays targeting the nucleoprotein gene of TOSV since we chose real-time tests for the detection of TOSV already available in the literature. It would be extremely useful to combine different tests targeting the three segments of the viral genome in a unique multi-region assay. It provides a robust assay that may be used to follow Dr. Meagan Kay’s recommendations (US CDC) *“…TOSV should be included in the differential list of viral pathogens among patients who seek treatment with symptoms consistent with meningitis or encephalitis if the patients have recently traveled to Mediterranean areas“* [32].

## 5. Conclusions

The development of a new molecular test for the detection of TOSV can encourage physicians to consider TOSV infection in cases of patients with neurological manifestations, as well as to implement entomological investigations on sandflies. The objective is to increase the awareness about this virus among researchers and physicians and to better characterize its circulation level in vectors and humans. 

To conclude, we proved that combining three monoplex systems in a Trio TOSV RT-qPCR is a simple way to have a more robust assay, reducing the risk of false-negative results. Moreover, the specificity and the sensitivity of monoplex assays are not impaired when combined in the multi-region assay; therefore, its use is recommended for both routine diagnosis and research projects. Further genetic studies will provide a better comprehension of genetic diversity of TOSV strains, especially concerning lineage C. This future characterization will help to optimize our molecular assay.

## Figures and Tables

**Table 1:** Primers and probes included in the Trio TOSV RT-qPCR assay.

Reference	Primer/Probe	5’→3’ Sequence	Target	Position ^a^	Amplicon Size (nts)	Concentration [nM]
Pérez-Ruiz et al. [23]	STOS-F	TGCTTTTCTTGATGAGTCTGCAG	S RNA, N gene	1718–1736	90	1000
	STOS-R	CAATGCGCTTYGGRTCAAA		1785–1807		1000
	STOS-P	FAM-ATCAATGCATGGGTRAATGAGTTTGCTTACC-TAMRA		1742–1772		200
Weidmann et al. [22]	TOS F	GGGTGCATCATGGCTCTT	S RNA, N gene	1381–1398	151	500
	TOS R	GCAGRGACACCATCACTCTGTC		1510–1531		500
	TOS P	FAM-CAATGGCATCCATAGTGGTCCCAGA-TAMRA		1415–1439		200
Brisbarre et al. [21]	TOS-IMT-F	TCTCCCAGGAAATGACATCC	S RNA, N gene	621–640	85	400
	TOS-IMT-R	AGATGGGWGTCTCTGGTCAT		706–725		400
	TOS-IMT-P	FAM-TGTGGTYCAAGCAGCACGGGTG-TAMRA		654–675		200

^a^ Refers to the sequence of TOSV strain 181135-14 (GenBank accession number KU573066).

**Table 2:** Protocols used for the evaluation of clinical samples using two different PCR master mixes.

**PCR master mix: qScript™ XLT One-Step RT-qPCR (Quanta Bio, VWR International Eurolab S.L., Llinars del Vallès, Spain)**					
Primers and probe: **P&P Trio TOSV assay**			Primers and probe: **P&P Perez-Ruiz monoplex assay**		
**Reagents and conditions** (as recommended by QuantaBio)			**Reagents and conditions** (as recommended by QuantaBio)		
Quanta mix (2×)	15 µL		Quanta mix (2×)	15 µL	
P&P Trio TOSV	7 µL		P&P TOSV	6 µL	
RNA	8 µL		RNA	8 µL	
			Water PCR grade	1 µL	
Total	30 µL		Total	30 µL	
**Amplification protocol**			**Amplification protocol**		
50 °C	10 min		50 °C	10 min	
95 °C	1 min		95 °C	1 min	
95 °C	10 s	45×	95 °C	10 s	45×
60 °C	30 s		60 °C	30 s	
**PCR master mix: Real-time ready RNA virus master (Roche Diagnostics, Barcelona, Spain)**					
Primers and probe: **P&P Trio TOSV assay**			Primers and probe: **P&P Perez-Ruiz monoplex assay**		
**Reagents and conditions** (as recommended by Roche)			**Reagents and conditions** (as recommended by Roche)		
RT-qPCR mix (5×)	4 µL		RT-qPCR mix (5×)	4 µL	
Enzyme	0.1 µL		Enzyme	0.1 µL	
P&P Trio TOSV	4.6 µL		P&P TOSV	4.6 µL	
RNA	5 µL		RNA	5 µL	
Water PCR grade	6.3 µL		Water PCR grade	6.3 µL	
Total	20 µL		Total	20 µL	
**Amplification protocol**			**Amplification protocol**		
50 °C	10 min		50 °C	10 min	
95 °C	30 s		95 °C	30 s	
95 °C	5 s	45×	95 °C	5 s	45×
60 °C	30 s		60 °C	30 s	

**Table 3:** Comparison of analytical sensitivity of Trio TOSV RT-qPCR assay with each of the three parental assays.

	LOD95: 23.8 copies/µL				LOD95: 11.3 copies/µL				LOD95: 20.6 copies/µL				LOD95: 84 copies/µL			
**Standard RNA**	**Trio**				**STOS Pérez-Ruiz**				**TOS2 Weidmann**				**IMT Brisbarre**			
RNA copies/µL	tested	positive	%	Ct value (SD)	tested	positive	%	Ct value (SD)	tested	positive	%	Ct value (SD)	tested	positive	%	Ct value (SD)
718	12	12	100	32.9 (0.4)	12	12	100	34.3 (0.3)	12	12	100	34.4 (0.2)	12	12	100	34.9 (0.5)
144	12	12	100	34.5 (0.7)	12	12	100	36.4 (1.1)	12	12	100	37.3 (0.7)	12	12	100	36.9 (0.5)
29	12	12	100	36.2 (0.3)	12	12	100	38.1 (0.7)	12	12	100	38.8 (0.4)	12	4	33	38.2 (0.8)
6	12	6	50	37.3 (1.4)	12	10	83	39.1 (0.5)	12	8	67	39.3 (0.8)	12	0	0	-
1	12	1	8	38.2	12	3	25	39.1 (0.8)	12	3	25	39.4 (0.5)	12	0	0	-
0	12	0	0	-	12	0	0	-	12	0	0	-	12	0	0	-
	LOD95: 21.9 copies/µL				LOD95: 19.1 copies/µL				LOD95: 19.2 copies/µL				LOD95: 101.1 copies/µL			
**TOSV A**	**Trio**				**STOS Pérez-Ruiz**				**TOS2 Weidmann**				**IMT Brisbarre**			
Viral RNA copies/µL	tested	positive	%	Ct value (SD)	tested	positive	%	Ct value (SD)	tested	positive	%	Ct value (SD)	tested	positive	%	Ct value (SD)
850	12	12	100	32.8 (0.3)	12	12	100	33.5 (0.5)	12	12	100	34.7 (0.6)	12	12	100	33.8 (0.2)
170	12	12	100	34.9 (0.4)	12	12	100	36.0 (1)	12	12	100	37.2 (0.7)	12	12	100	35.9 (0.5)
34	12	12	100	36.6 (0.8)	12	12	100	37.7 (0.9)	12	12	100	39.0 (0.4)	12	2	17	38.7 (0.6)
7	12	2	17	38.3 (0.6)	12	9	75	38.1 (0.7)	12	3	25	39.4 (0.2)	12	0	0	-
1	12	0	0	-	12	3	25	38.5 (0.8)	12	0	0	-	12	0	0	-
0	12	0	0	-	12	0	0	-	12	0	0	-	12	0	0	-
	LOD95: 22.3 copies/µL				LOD95: 14.9 copies/µL				LOD95: 18.3 copies/µL				LOD95: 93.3 copies/µL			
**TOSV B**	**Trio**				**STOS Pérez-Ruiz**				**TOS2 Weidmann**				**IMT Brisbarre**			
Viral RNA copies/µL	tested	positive	%	Ct value (SD)	tested	positive	%	Ct value (SD)	tested	positive	%	Ct value (SD)	tested	positive	%	Ct value (SD)
1075	12	12	100	31.9 (0.4)	12	12	100	32.5 (0.4)	12	12	100	33.3 (0.4)	12	12	100	33.2 (0.2)
215	12	12	100	34.1 (0.3)	12	12	100	35.1 (0.3)	12	12	100	35.7 (0.7)	12	12	100	36.1 (0.4)
43	12	12	100	37.1 (0.9)	12	12	100	37.6 (1.0)	12	12	100	37.9 (0.5)	12	5	42	38.8 (1)
9	12	4	33	38.6 (0.4)	12	10	83	39.0 (0.7)	12	6	50	38.4 (0.4)	12	0	0	-
2	12	0	0	-	12	2	17	39.3 (0.9)	12	0	0	-	12	0	0	-
0	12	0	0	-	12	0	0	-	12	0	0	-	12	0	0	-

## Data Availability

All data are included in the article and Supplementary Material.

## Supplementary Materials

The following are available online at https://www.mdpi.com/2076-0817/10/3/254/s1, Supplementary File 1. Sequence of RNA Synthetic Transcript (standard RNA) containing the three regions targeted by the three monoplex RT-qPCR assays included in the Trio TOSV assay. For each region, a sequence not present in wild-type virus (Not1, in blue) was included allowing to identify contamination caused by the positive control. Supplementary File 2. Strains tested to assess specificity of Trio Toscana virus (TOSV) RT-qPCR assay. Supplementary File 3. RT-qPCR results observed on TOSV RNAs obtained from 64 CSF samples tested with Trio TOSV assay and Pérez-Ruiz monoplex assay [16], using two different Master mixes.
