# Supplementary material for: Evaluation of a Trio Toscana Virus Real-Time RT-PCR Assay Targeting Three Genomic Regions within Nucleoprotein Gene
Source: Pathogens. 2021 Feb 24;10(3):254. doi: 10.3390/pathogens10030254 (PMC7996202; doi:10.3390/pathogens10030254)
Supplement: Supplementary file 1 [file pathogens-10-00254-s001.zip › pathogens-996164-supplementary.pdf]

Supplementary file

Supplementary file 1. Sequence of RNA Synthetic Transcript (standard RNA) containing the three regions targeted by the three monoplex RT-qPCR assays included in the Trio TOSV assay. For each region, a sequence not present in wild-type virus (Not1, in blue) was included allowing to identify contamination caused by the positive control.

GGGTGCATCATGGCTCTTGGGTAGGTCACGCCGGCAATGGCATCCATAGTGGTCCCAGAAACT
GGCAGTGATTCTGAATTATAGCGGCCGCTTATTACGGTCCATGGAACAAAAGCAGCCGACAC
TCTAGACAGAGTGATGGTGTCTCTGCCAATGCGCTTTGGGTCAAACCCTTGGTAAGCAAACTC
ATTCACCCATGCATTGATATTATAGCGGCCGCTTATTACTGCAGACTCATCAAGAAAAGCATC
TCCCAGGAAATGACATCCTCGGAGACATCTGTGTGGTTCAAGCAGCGCGGGTGACAAGATTA
TAGCGGCCGCTTATTACCAAGATGACCAGAGACACCCATCT

Supplementary file 2. Strains tested to assess specificity of Trio Toscana virus (TOSV) RT-qPCR assay.

Taxonomy Family, Genus	Viruses and acronym		Strain	Viral load $TCID_{50}$/mL	Reference on EVAg or NCPV catalogue
Phenuiviridae Phlebovirus	Toscana virus	TOSV-lineage A	UVE/TOSV/2014/FR/5904	$10^{8,22}$	001V-02452 (EVAg)
		TOSV-lineage B	UVE/TOSV/2013/FR/113	$10^{7,42}$	001V-02461 (EVAg)
	Adana virus	ADAV	UVE/ADAV/2012/TR/195	$10^{7,82}$	001V-02364 (EVAg)
	Arbia virus	ARBV	UVE/ARBV/2010/TN/Tunisie2010 T91	$10^{7,57}$	001V-02366 (EVAg)
	Corfou virus	CFUV	UVE/CFUV/UNK/GR/PaAr 814	$10^{6,49}$	001V-02367 (EVAg)
	Dashli virus	DASV	UVE/DASV/2011/IR/90	$10^{2,42}$	001V-02368 (EVAg)
	La Crosse virus	LACV	-	$10^{6,82}$	0005071V (NCPV)
	Massilia virus	MASV	UVE/MASV/2009/FR/M43	$10^{7,22}$	001V-02369 (EVAg)
	Medjerda Valley virus	MVV	UVE/MVV/2010/TN/T131	$10^{7,42}$	001V-02379 (EVAg)
	Punique virus	PUNV	UVE/PUNV/2008/TN/P1_B4_2008	$10^{6,32}$	001v-EVA152 (EVAg)
	Rift Valley Fever virus	RVFV	UVE/RVFV/UNK/XX/Smithburn vaccine	$10^{7,32}$	001V-02385 (EVAg)
	Sandfly fever Naples virus	SFNV	UVE/SFNV/UNK/IT/30451	$10^{4,57}$	001V-02386 (EVAg)
	Sandfly fever Sicilian virus	SFSV	UVE/SFSV/1943/IT/Sabin	$10^{6,82}$	001v-EVA77 (EVAg)
	Tehran virus	TEHV	UVE/THEV/1960/IR/I-47	$10^{2,82}$	001V-02387 (EVAg)

	Toros virus	TORV	UVE/TORV/2012/TR/213	$10^{3,42}$	001V-02424 (EVAg)
	Zerdali virus	ZERV	UVE/ZERV/2013/TR/37	$10^{6,42}$	001V-02117 (EVAg)
	Uukuniemi virus	UUKV	UVE/UUKV/UNK/XX/TC259	$10^{4,57}$	001V-02463 (EVAg)
Flaviviridae Flavivirus	Japanese encephalitis virus	JEV	UVE/JEV/2009/LA/CNS769	$10^{5.57}$	001V-02217 (EVAg)
	Saint-Louis encephalitis virus	SLEV	UVE/SLEV/UNK/US/MSI-7	$10^{4,82}$	001v-EVA128 (EVAg)
	Tick-borne encephalitis virus	TBEV	UVE/TBEV/2013/FR/32.11 WT-PCR	$10^{8,82}$	001V-02352 (EVAg)
	Yellow fever virus	YFV	UVE/YFV/UNK/XX/French neurotropic	$10^{7,32}$	001V-02226 (EVAg)
	West Nile virus	WNV	UVE/WNV/2008/US/R94224	$10^{7,32}$	001V-02224 (EVAg)
	Usutu virus	USUV	UVE/USUV/1959/ZA/SAAR-1776	$10^{5,32}$	001v-EVA138 (EVAg)
	Murrey Valley encephalitis virus	MVEV	UVE/MVEV/UNK/AU/3329	$10^{4,32}$	001v-EVA145 (EVAg)
Togaviridae Alphavirus	Venezuelan equine encephalitis virus	VEEV	UVE/VEEV/UNK/XX/TC83 vaccine	$10^{9,42}$	001v-EVA1459 (EVAg)
	Western equine encephalitis virus	WEEV	UVE/WEEV/UNK/XX/47a	$10^{8,32}$	001v-EVA1479 (EVAg)
	Eastern equine encepahlitis virus	EEEV	UVE/EEEV/1999/XX/H178_99	$10^{7,82}$	001v-EVA1480 (EVAg)
	O'nyong-nyong virus	ONNV	UVE/ONNV/UNK/SN/Dakar 234	$10^{4,22}$	001v-EVA1044 (EVAg)
	Chikungunya virus	CHIKV	UVE/CHIKV/2017/FR/45625-26	$10^{6,16}$	001V-03433 (EVAg)
	Semliki Forest virus	SFV	UVE/SFV/UNK/XX/1745	$10^{4,42}$	001V-02468 (EVAg)
	Sindbis virus	SINV	UVE/SINV/UNK/EG/Egypt 339	$10^{4,32}$	001V-02469 (EVAg)
Picornaviridae Enterovirus	Human echovirus 30	EV30	UVE/E-30/2013/FR/7303	$10^{6,42}$	001V-02522 (EVAg)
	Coxsackievirus B3	CVB3	UVE/CV-B3/1993/FR/2679	NA (30 ct)	001v-EVA367 (EVAg)
	Human enterovirus 71	EV71	UVE/EV-A71/2011/LA/HFMD18TS	$10^{6,07}$	001v-EVA1553 (EVAg)
Herpesviridae Simplexvirus	Herpes simplex virus 1	HSV1	UVE/HHV-1/2007/FR/7351	10 6,32	001v-EVA961 (EVAg)

	Herpes simplex virus 2	HSV2	UVE/HHV-2/2005/FR/8724	10 5,32	001v-EVA962 (EVAg)
	Varicella zoster virus	VZV	Clinical sample	NA (10 ct)	-

Supplementary file 3. RT-qPCR results observed on TOSV RNAs obtained from 64 CSF samples tested with Trio TOSV assay and Pérez-Ruiz monoplex assay [16], using two different Master mixes.

	qScript™ XLT One-Step RT-qPCR (QuantaBio)			RealTime ready RNA Virus Master (Roche Diagnostics)		
**Sample ID**	**Ct value (Trio TOSV assay)**	**Ct value (Pérez-Ruiz monoplex assay)**	**ΔCt value (Ct Trio TOSV-Ct Pérez-Ruiz monoplex assay)**	**Ct value (Trio TOSV assay)**	**Ct value (Pérez-Ruiz monoplex assay)**	**ΔCt value (Ct Trio TOSV-Ct Pérez-Ruiz monoplex assay)**
#1	28.78	31.43	-2.65	32.3	32.83	-0.53
#2	26.1	28.76	-2.66	30.38	31.22	-0.84
#3	23.27	27.48	-4.21	29.2	30.2	-1.00
#4	24.78	26.25	-1.47	29.12	29.2	-0.08
#5	25.61	29.1	-3.49	NA	NA	NA
#6	ND	ND	ND	ND	ND	ND
#7	22.64	24.08	-1.44	27.14	26.85	0.29
#8	25.22	26.23	-1.01	29.17	28.72	0.45
#9	22.81	24.79	-1.98	27.12	27.31	-0.19
#10	23.23	26.2	-2.97	NA	NA	NA
#11	22.93	24.86	-1.93	27.14	27.69	-0.55
#12	24.57	27.42	-2.85	30.43	31.46	-1.03
#13	25.08	26.5	-1.42	29.44	29.72	-0.28
#14	23.41	24.87	-1.46	28.01	28.11	-0.10
#15	29.8	31.35	-1.55	33.82	33.35	0.47
#16	25.7	28.06	-2.36	30.07	30.52	-0.45
#17	24.87	27.54	-2.67	29.52	29.56	-0.04
#18	23.57	28.22	-4.65	30.14	30.29	-0.15
#19	24.08	26.86	-2.78	29.02	29.34	-0.32
#20	25.59	27.34	-1.75	29.84	31.06	-1.22
#21	25.11	29.06	-3.95	31.25	31.89	-0.64
#22	25.98	28.93	-2.95	31.28	31.45	-0.17
#23	22.63	22.76	-0.13	26.92	26.61	0.31
#24	25.24	24.43	0.81	NA	NA	NA
#25	24.39	25.11	-0.72	NA	NA	NA
#26	27.88	29.2	-1.32	NA	NA	NA
#27	23.85	24.29	-0.44	28	28.08	-0.08
#28	23.68	25.33	-1.65	27.64	28.02	-0.38
#29	24.58	32.36	-7.78	29.45	NA	
#30	21.95	22.62	-0.67	26.73	26.34	0.39
#31	23.31	25.48	-2.17	29.08	29.22	-0.14

#32	ND	ND	ND	NA	NA	NA
#33	24.37	27	-2.63	29.08	29.4	-0.32
#34	24.61	29.12	-4.51	30.69	NA	
#35	22.5	23.16	-0.66	27.05	NA	
#36	ND	ND	ND	ND	ND	ND
#37	28.67	31.63	-2.96	33.57	33.93	-0.36
#38	27.96	31.21	-3.25	NA	NA	NA
#39	24.04	26.25	-2.21	NA	NA	NA
#40	25.53	27.1	-1.57	29.98	30.45	-0.47
#41	22.28	24.27	-1.99	26.58	26.55	0.03
#42	23.3	24.98	-1.68	27.1	27.62	-0.52
#43	24.11	24.97	-0.86	27.56	27.94	-0.38
#44	24.64	24.41	0.23	NA	NA	NA
#45	24.07	26.11	-2.04	29.27	29.33	-0.06
#46	26.81	29.88	-3.07	32.61	33.05	-0.44
#47	26.98	28.48	-1.50	30.35	30.77	-0.42
#48	25.97	27.84	-1.87	28.77	29.12	-0.35
#49	25.92	27.57	-1.65	29.88	30.24	-0.36
#50	28.09	28.65	-0.56	30.58	30.13	0.45
#51	27.65	28.53	-0.88	30.6	30.77	-0.17
#52	44.54	31.86	12.68	34.59	34.73	-0.14
#53	41.1	28.35	12.75	36.55	33.24	3.31
#54	24.17	27.35	-3.18	28.88	30.31	-1.43
#55	29.34	31.01	-1.67	33.09	33.57	-0.48
#56	25.48	26.67	-1.19	28.94	29.97	-1.03
#57	25.35	26.18	-0.83	28.29	27.75	0.54
#58	26.06	28.04	-1.98	30.03	30.49	-0.46
#59	26.77	29.36	-2.59	31.37	31.33	0.04
#60	25.61	27.76	-2.15	30.29	29.82	0.47
#61	23.2	24.75	-1.55	NA	NA	NA
#62	23.8	24.56	-0.76	27.39	27.67	-0.28
#63	28.18	30.18	-2.00	32.27	32.3	-0.03
#64	26.84	29.01	-2.17	30.37	31.1	-0.73

ND: not detected. NA: insufficient sample volume to perform molecular test.

~~**Supplementary file 3.** Sequence of RNA Synthetic Transcript (standard RNA) containing the three regions targeted by the three monoplex RT-qPCR assays included in the Trio TOSV assay. For each region, a sequence not present in wild-type virus (Not1, in blue) was included allowing to identify contamination caused by the positive control.~~

~~GGGTGCATCATGGCTCTTGGGTAGGTCACGCCGGCAATGGCATCCATAGTGGTCCCAGAAACT
GGCAGTGATTCTGAATTATAGCGGCCGCTTATTACGGTCCATGGAACAAAAGCAGCCGACAC
TCTAGACAGAGTGATGGTGTCTCTGCCAATGCGCTTTGGGTCAAACCCTTGGTAAGCAAACTC
ATTCACCCATGCATTGATATTATAGCGGCCGCTTATTACTGCAGACTCATCAAGAAAAGCATC
TCCCAGGAAATGACATCCTCGGAGACATCTGTGTGGTTCAAGCAGCGCGGGTGACAAGATTA
TAGCGGCCGCTTATTACCAAGATGACCAGAGACACCCATCT~~
